# 

**Published:** 1872-12-01

**Authors:** 


Money Receipts to November 1st, 1872.—Dr's. D. Lord, $3.00; W. S. Robertson, $3.00; D. T. Nelson, $3.00; A. J. Smith, $3.00; Chas. Wirth, $3.00; D. Newell. $6.00; M. Parker, $6.00; J. Milleron, $6.00; Chester Reeder, $15.00; B. F. Rolfe, $3.00; J. T. Frazer, $3.00; W. M. Burbank, $3.00; D. A. McDonald, $3.00; M. A. Seamon, $10.00.

